# Remarks of Dr. E. E. Cruzen

**Published:** 1898-06

**Authors:** 


### REMARKS OF DR. E. E. CRUZEN.

(Omitted in proceedings.)

*Gentlemen:* The Association of Dental Surgeons is an effort in the right direction, and to my mind a success. It has long been my idea that if we would progress in our profession, we should with a feeling of fellowship be bonded together in our association for the discussion of the things pertaining to dentistry. Practicing as I have for ten years in a place where I was denied the pleasure of meeting with my fellow dentists in an association except on my visits to Baltimore at annual meeting of State Association, I felt honored and much pleased when an invitation was extended me to become a member of the Association of Dental Surgeons shortly after I had adopted Baltimore as my home, and I can assure you it is with pleasure I look forward to our monthly meetings, for to me they have been profitable as well as has been pleasant the social intercourse with my brother dentists. The first meeting it was my privilege to attend was at the office of our honored President, and that meeting to me was a treat indeed, for I recognized the artist of rare ability in the repousse work I saw there which had been wrought by the hands of our guest of this evening.

